# The habit of drinking and driving in Brazil: National Survey of Health 2013 and 2019

**DOI:** 10.11606/s1518-8787.2022056004472

**Published:** 2022-11-18

**Authors:** Lucas Sisinno Ribeiro, Giseli Nogueira Damacena, Paulo Roberto Borges de Souza, Célia Landmann Szwarcwald

**Affiliations:** I Fundação Oswaldo Cruz Instituto de Comunicação e Informação Científica e Tecnológica em Saúde Programa de Pós-Graduação em Informação e Comunicação em Saúde Rio de Janeiro RJ Brasil Fundação Oswaldo Cruz. Instituto de Comunicação e Informação Científica e Tecnológica em Saúde. Programa de Pós-Graduação em Informação e Comunicação em Saúde. Rio de Janeiro, RJ, Brasil; II Fundação Oswaldo Cruz Instituto de Comunicação e Informação Científica e Tecnológica em Saúde Laboratório de Informações em Saúde Rio de Janeiro RJ Brasil Fundação Oswaldo Cruz. Instituto de Comunicação e Informação Científica e Tecnológica em Saúde. Laboratório de Informações em Saúde. Rio de Janeiro, RJ, Brasil

**Keywords:** Accidents, Traffic, prevention & control, Driving Under the Influence, Binge Drinking, Risk Factors, Health Surveys

## Abstract

**OBJECTIVE:**

To assess factors associated with the habit of drinking and driving and estimating the variations in the prevalence of this behavior in 2013 and 2019, considering information from the two editions of the *Pesquisa Nacional de Saúde* (PNS – National Survey of Health).

**METHODS:**

PNS is a nationwide cross-sectional home-based study. In 2013 and 2019, 60,202 and 85,854 individuals were interviewed, respectively. To assess the association between the indicator “drinking and driving” and the study variables, crude and adjusted odds ratios (ORs) were estimated using logistic regression models. To compare the prevalence between the studied years, a Pearson’s chi-squared test adjusted by the Rao-Scott correction (which considers the effect of the sampling plan) and converted into an F statistic, tested at a 5% significance level, was used.

**RESULTS:**

The prevalence of drinking and driving was higher among men in 2013 (27.4%; 95%CI 25.6–29.3%) and 2019 (20.5%; 95%CI 19.4–21.7%) than among women (11.9%; 95%CI 9.9–14.2% and 7.2%; 95%CI 6.7–9.0%, respectively). Inidviduals aged 30 to 39, who lived without a partner, in rural areas, and were motorcycle drivers had significantly higher estimates. Men with higher income had higher prevalence of drinking and driving. From 2013 to 2019, the act of drinking and driving significantly decreased. Regarding traffic accidents, ORs were significant (p < 0.01) in the studied years for both men and women.

**DISCUSSION:**

Results show the need to continue policies to monitor blood alcohol level and traffic education, with specific actions directed to rural areas and motorcycle drivers.

## INTRODUCTION

Alcohol is a widely spread licit drug that has been used in different cultures for many centuries. It is associated with festivities, religious ceremonies, and celebrations^1^. Due to its psychotropic characteristics, its use favors sociability and integration among individuals and is currently a common and frequent social habit. However, although alcohol is a licit drug, certain consumption patterns can cause harmful consequences for the health of individuals and for society^2^.

Mental and behavioral disorders, including dependence, chronic non-communicable diseases, such as liver cirrhosis, some types of cancer, and cardiovascular diseases, and injuries resulting from violence and traffic accidents are some problems associated with alcohol abuse^3^. According to the World Health Organization (WHO), harmful alcohol consumption is associated with more than three million deaths per year worldwide^4^. In Brazil, data from the Global Burden of Diseases (GBD) show that in 2017, alcohol consumption ranked 5th among the risk factors that most contribute to the total number of disability-adjusted years of life lost^5,6^.

Considering the effects of alcohol in the body, driving after its consumption is considered one of the main causes of traffic accidents^5^. Sensory modifications caused by alcohol intoxication compromise the psychomotor skills of individuals, causing risks to the driver, passengers, and pedestrians^1,7^.

According to the WHO Global Health Observatory, in 2019, Brazil had an estimated traffic mortality rate of 16.0 (per 100,000 inhabitants), which is much higher than that of developed countries, such as Australia (4.9 per 100,000 inhabitants) and Canada (5.3 per 100,000 inhabitants) but close to that of other emerging countries, such as China (17.4 per 100,000 inhabitants)^8^. Thus, monitoring blood alcohol level plays is essential to prevent traffic accidents in the country^5,2^.

In the United States, data from the National Highway Traffic Safety Administration showed that in 2016, 10,497 people died in traffic accidents caused by alcohol, which represented 28% of all traffic-related deaths^9^. In Brazil, information from the *Departamento Nacional de Infraestrutura de Transportes* (DNIT – National Department of Transportation Infrastructure) showed that the Federal Highway Police, in 2017, caught 19,083 individuals driving after consuming alcohol and in the same year, 6,450,000 (33.8%) accidents on federal highways were caused by drunk drivers, resulting in 13,000 victims and about 1,000 deaths^10^.

A study based on data from the *Pesquisa Nacional de Saúde* (PNS – National Survey of Health) 2013 showed that 24.4% of Brazilians had the habit of drinking and driving; this behavior was associated with a higher risk of traffic accidents and men aged 18 to 39 years had the highest risk of driving under the influence of alcohol^2^.

Data from the *Vigilância de Fatores de Risco e Proteção para Doenças Crônicas por Inquérito Telefônico* (Vigitel – Surveillance System for Risk and Protective Factors for Chronic Diseases by Telephone Survey) in the same year showed that 29.3% of men and 16.5% of women living in capital municipalities had the habit of drinking and driving^1^. A recent study with Vigitel data showed a decrease in the indicator of drinking and driving from 2007 to 2018^5^.

In the second edition of the PNS (2019), questions related to the habit of drinking and driving were repeated, making it possible to estimate the temporal variation of this behavior in the Brazilian population.

This study aimed to assess factors associated with drinking and driving and estimating the variations in the prevalence of this behavior in 2013 and 2019, considering information from the two editions of the PNS.

## METHODS

This is a cross-sectional study based on data from the 2013 and 2019 editions of the PNS.

The PNS is a home-based and nationwide research held by the Brazilian Ministry of Health in partnership with the Brazilian Institute of Geography and Statistics (IBGE) in 2013 and 2019. The PNS was approved by the *Comissão Nacional de Ética em Pesquisa* (Conep – National Research Ethics Commission) in July 2013, under no. 328,159, and in August 2019, under no. 3,529,376.

### Sampling

The studied sample included residents of permanent private households in Brazil, except for those located in special census tracts.

The PNS sample was a subsample of the *Amostra Mestra do Sistema Integrado de Pesquisas Domiciliares* (SIPD – Master Sample of Integrated Household Surveys) of IBGE. The sampling plan was carried out by conglomerates in three selection stages (census tracts or sector composition, households, and individuals) with stratification of the primary sampling units (PSU). Souza Junior detailed the sampling process and the calculation of expansion factors^11^.

In 2013, 60,202 individuals aged 18 years or older were selected for an individual interview; in 2019, this number was 85,854.

### Study Variables

Residents aged 18 years or older selected in the household answered an individual questionnaire.

For the construction of the indicator “drinking and driving,” the following question was used in the two editions of the PNS: “On any of these days when you consumed alcohol, did you drive right after drinking?” Individuals could answer: 1) Yes; 2) No. Only individuals who consumed alcohol and drove a car or motorcycle were asked this question. Drivers were identified from the following questions: “Do you currently drive a car (including taxi, transport apps, or others)?” and “Do you currently drive a motorcycle?” (Figure). This indicator was analyzed according to the following sociodemographic characteristics: sex (men or women); age group (18–29 years, 30–39 years, or ≥ 40 years); skin color/ethnicity (white or non-white); married or living with a partner (yes or no); *per capita* income (< 1 minimum wage or ≥ 1 minimum wage); residence status (urban or rural); and type of vehicle driven (only car or motorcycle).

**Figure:**
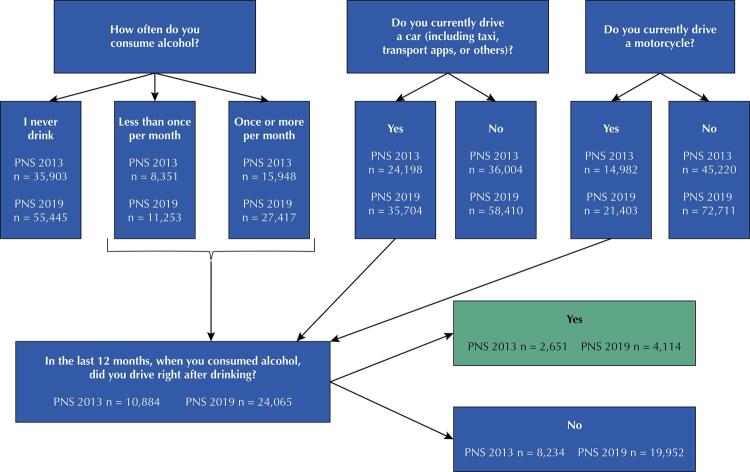
Flow of questions to identify individuals who had the habit of drinking and driving. PNS, Brazil, 2013 and 2019.

### Data Analysis

For each category of the variables studied, the prevalence of the indicator “drinking and driving” and their respective 95% confidence intervals were estimated in 2013 and 2019. To assess the association between the indicator “drinking and driving” and the study variables, crude and adjusted odds ratios (ORs) were estimated using logistic regression models.

To compare the prevalence in 2013 and 2019, a Pearson’s chi-squared test adjusted by the Rao-Scott correction (which considers the effect of the sampling plan) and converted into an F statistic, tested at a 5% significance level, was used.

Moreover, the involvement of individuals in traffic accidents in the last 12 months as car, van, or motorcycle drivers, depending on the sex, with bodily injuries was assessed with the following question: “In the last 12 months, have you been involved in a traffic accident in which you suffered bodily injuries?” To assess the association between the indicator “drinking and driving” and the involvement in traffic accidents in the last 12 months, logistic regression models by sex were used. Crude and adjusted ORs were estimated by the variables age group, married or living with a partner, and *per capita* income, with a 5% significance level.

The statistical analysis considered the sample design of the two PNS, including expansion factors and conglomeration effects. Data were analyzed using the Software for Statistics and Data Science (Stata)^12^ version 14.0, “survey” module.

## RESULTS

We analyzed 2,651 individuals in 2013 and 4,114 in 2019 who drove after drinking. In both studied years, the prevalence of drinking and driving was higher among men (27.4%, 95%CI: 25.6–29.3% in 2013; and 20.5%, 95%CI: 19.4–21.7% in 2019) than among women (11.9%, 95%CI: 9.9–14.2% in 2013; and 7.2%, 95%CI: 6.7–9.0% in 2019) (Tables 1 and 2). This behavior was more prevalent among both men and women aged 30 to 39 years in both editions of the PNS.

**Table 1:** Prevalence of the habit of drinking and driving and odds ratios (crude and adjusted) among men aged 18 years or older, according to sociodemographic variables, residence status, and the type of vehicle driven. PNS, Brazil, 2013 and 2019.

Variables		2013										2019									
	
		n	%	95%CI		OR	95%CI		OR_adj_	95%CI		n	%	95%CI		OR	95%CI		OR_adj_	95%CI	
		
				LL	UL		LL	UL		LL	UL			LL	UL		LL	UL		LL	UL
Total		2,397	27.4	25.6	29.3	-	-	-	-	-	-	3,539	20.5	19.4	21.7	-	-	-	-	-	-
Age group	18–29	779	29.7	26.3	33.2	1.39ª	1.13	1.70	1.29^a^	1.02	1.63	966	23.5	21.2	26.0	1.51ª	1.28	1.80	1.44^a^	1.20	1.73
	30–39	740	31.4	28.0	35.1	1.51ª	1.22	1.86	1.45^a^	1.17	1.80	1,123	24.7	22.4	27.2	1.62ª	1.35	1.93	1.56^a^	1.30	1.87
	≥ 40	878	23.3	20.9	25.9	1.00	-	-	1.00	-	-	1,449	16.9	15.3	18.6	1.00	-	-	1.00	-	-
Ethnicity/ skin color	White	1,191	25.5	23.2	28.1	1.00	-	-	1.00	-	-	1,574	18.9	17.4	20.5	1.00	-	-	1.00	-	-
	Non-white	1,188	29.9	27.2	32.6	1.24^a^	1.05	1.47	1.24^a^	1.04	1.49	1,965	22.2	20.4	24.0	1.22^a^	1.06	1.42	1.16	0.99	1.37
Married or living with a partner	Yes	1,005	25.4	23.0	28.0	1.00	-	-	1.00	-	-	1,554	18.8	17.1	20.7	1.00	-	-	1.00	-	-
	No	1392	29.1	26.6	31.7	1.21ª	1.02	1.42	1.07	0.88	1.28	1,985	22.1	20.7	23.6	1.22ª	1.06	1.41	1.03	0.88	1.21
Per capita income	< 1 MW	764	24.5	21.7	27.5	1.00	-	-	1.00	-	-	1,360	20.1	18.5	21.8	1.00	-	-	1.00	-	-
	≥ 1 MW	1,631	29.0	26.8	31.4	1.26ª	1.05	1.51	1.57^a^	1.28	1.92	2,179	20.8	19.3	22.4	1.05	0.92	1.19	1.35^a^	1.15	1.57
Residence status	Urban	2,063	26.7	24.7	28.8	1.00	-	-	1.00	-	-	2,901	19.8	18.5	21.1	1.00	-	-	1.00	-	-
	Rural	333	33.1	28.9	37.6	1.36ª	1.09	1.69	1.47^a^	1.16	1.86	638	25.0	22.9	27.3	1.36ª	1.18	1.56	1.34^a^	1.15	1.57
Type of vehicle driven	Only car	860	24.3	21.6	27.1	1.00	-	-	1.00	-	-	1,421	17.6	16.2	19.1	1.00	-	-	1.00	-	-
	Motor-cycle	1,537	29.5	27.2	32.1	1.31^a^	1.09	1.57	1.20	0.98	1.48	2,118	23.1	21.5	24.8	1.40^a^	123	1.60	1.28^a^	1.09	1.51

95%CI: 95% confidence interval; LL: lower limit of the confidence interval; UL: upper limit of the confidence interval; MW: minimum wage; OR: crude odds ratio; OR_adj_: odds ratio adjusted for other variables.

^a^ p ≤ 0.05.

**Table 2:** Prevalence of the habit of drinking and driving and odds ratios (crude and adjusted) among women aged 18 years or older, according to sociodemographic variables, residence status, and the type of vehicle driven. PNS, Brazil, 2013 and 2019.

Variables		2013										2019									
	
		n	%	95%CI		OR	95%CI		OR_adj_	95%CI		n	%	95%CI		OR	95%CI		OR_adj_	95%CI	
					
				LL	UL		LL	UL		LL	UL			LL	UL		LL	UL		LL	UL
Total		255	11.9	9.9	14.2	-	-	-	-	-	-	506	7.8	6.7	9.0	-	-	-	-	-	-
Age group	18–29	86	10.7	7.8	14.6	1.19	0.76	1.85	0.82	0.52	1.29	126	7.2	5.1	9.9	1.20	0.78	1.84	1.03	0.65	1.63
	30–39	103	16.3	12.3	21.4	1.92^a^	1.28	2.89	1.78^a^	1.15	2.77	212	10.8	8.6	13.4	1.89^a^	1.34	2.63	1.88^a^	1.32	2.69
	≤ 40	66	9.2	6.6	12.6	1.00	-	-	1.00	-	-	168	6.0	4.9	7.4	1.00	-	-	1.00	-	-
Ethnicity/ skin color	White	153	11.4	9.0	14.3	1.00	-	-	1.00	-	-	284	7.1	5.8	8.8	1.00	-	-	1.00	-	-
	Non-white	99	12.7	9.8	16.5	1.14	0.80	1.61	0.90	0.62	1.29	214	8.6	7.0	10.4	1.22	0.89	1.66	1.13	0.79	1.62
Married or living with a partner	Yes	66	8.2	5.5	11.9	1.00	-	-	1.00	-	-	129	5.2	3.9	7.1	1.00	-	-	1.00	-	-
	No	188	14.1	11.6	17.1	1.85^a^	1.20	2.84	2.09^a^	1.38	3.17	377	9.3	7.9	11.0	1.86^a^	1.28	2.70	1.94^a^	1.32	2.84
*Per capita* income	< 1 MW	65	12.1	8.8	16.5	1.00	-	-	1.00	-	-	142	7.9	6.1	10.1	1.00	-	-	1.00	-	-
	≥ 1 MW	189	11.8	9.5	14.5	0.97	0.67	1.40	1.28	0.84	1.95	364	7.7	6.5	9.2	0.98	0.70	1.37	1.24	0.87	1.78
Residence status	Urban	239	11.9	9.8	14.3	1.00	-	-	1.00	-	-	458	7.6	6.5	8.9	1.00	-	-	1.00	-	-
	Rural	15	11.6	6.4	20.2	0.98	0.66	1.43	0.92	0.59	1.44	48	9.5	6.4	13.9	1.28	0.80	2.04	1.25	0.77	2.05
Type of vehicle driven	Only car	132	10.0	7.7	12.9	1.00	-	-	1.00	-	-	318	7.4	6.2	9.0	1.00	-	-	1.00	-	-
	Motor-cycle	122	14.8	11.6	18.8	1.57^a^	1.08	2.27	1.68^a^	1.10	2.56	188	8.4	6.7	10.5	1.14	0.83	1.56	1.04	0.73	1.47

95%CI: 95% confidence interval; LL: lower limit of the confidence interval; UL: upper limit of the confidence interval; MW: minimum wage; OR: crude odds ratio; OR_adj_: odds ratio adjusted for other variables.

^a^ p ≤ 0.05.

In 2013, men aged 18 to 29 years and 30 and 39 years had ORs of 1.3 and 1.5, respectively, compared with those aged 40 years or more. In 2019, ORs were, respectively, 1.5 and 1.6, which made them significant even after adjustment for other variables (Table 1).

The habit of drinking and driving showed higher prevalence among non-white men. In 2013 and 2019, non-white men had a significantly higher chance of drinking and driving compared with white men. However, after controlling other variables, ORs were significant only in 2013 (Table 1).

In the two studied years, drinking and driving was more frequent among individuals who were not married or who did not live with a partner. The chance was 20% higher among men living without a partner, but ORs were not significant after adjustment for other variables (Table 1).

Regarding *per capita* income, the habit of drinking and driving was higher among men whose income is ≥ 1 minimum wage. Compared with men with income < 1 minimum wage, in 2013, crude and adjusted ORs were statistically significant (1.4 and 1.5, respectively). In 2019, only adjusted OR was significant, showing that, after age control, this behavior was more prevalent among men with higher *per capita* income (Table 1).

Regarding residence status (urban or rural), men living in the rural area had higher prevalence of drinking and driving. Compared with urban residents, both crude and adjusted ORs were significant in the two studied years (Table 1).

Regarding the type of vehicle driven, men who drove motorcycles had crude OR significantly > 1 compared with those who drove only car in the two studied years. However, after controlling for other variables, ORs were significant only in 2019 (Table 1).

Table 2 presents the results for women. The habit of drinking and driving was more prevalent among women aged 30 to 39 years in 2013 and 2019. Compared with women aged 40 years or older, crude and adjusted ORs were significant, ranging from 1.7 to 1.9. Compared with those aged 30 to 39 years, the results for women aged 18 to 29 years were not statistically significant.

Similarly to men, women who were not married or lived without a partner had the highest prevalence of drinking and driving. In 2013 and 2019, for these women, when compared with those living with a partner, crude ORs were 1.8 and adjusted ORs were 2.2 and 1.9, respectively, which made them significant at the 5% level (Table 2).

Regarding the type of vehicle driven, women who drove motorcycles had higher prevalence of drinking and driving only in 2013. In that year, compared with women who drove only car, crude and adjusted ORs were significant (1.6 and 1.7, respectively) (Table 2). We found no evident differences in the habit of drinking and driving according to skin color or *per capita* income (Table 2).

Table 3 shows the prevalence of this habit in 2013 and 2019. The decrease was significant: from 27.4% to 20.5% among men and from 11.9% to 7.8% among women. Regarding variations by age group, among men of all age groups, the decrease was significant; among women, the prevalence decreased among those aged 30 to 39 years and 40 years or older (Table 3). Regarding skin color, for both white and non-white men and women, the decrease was significant. The prevalence of drinking and driving also significantly decreased among individuals who were not married or lived without a partner. Among individuals who were married or lived with a partner, this decrease was significant only among men (Table 3). The analysis by *per capita* income showed a significant decrease in drinking and driving for the two categories among both men and women.

**Table 3:** Comparison between the prevalence of drinking and driving in 2013 and 2019, according to sociodemographic characteristics and the type of vehicle driven. PNS, Brazil, 2013 and 2019.

Variables		Men			Women		
	
		% 2013	% 2019	p^a^	% 2013	% 2019	p^a^
Total		27.4	20.5	< 0.001	11.9	7.8	< 0.001
Age group	18–29	29.7	23.5	0.003	10.7	7.2	0.080
	30–39	31.4	24.7	0.002	16.3	10.8	0.023
	≥ 40	23.3	16.9	< 0.001	9.2	6.0	0.031
Ethnicity/skin color	White	25.5	18.9	< 0.001	11.4	7.1	0.003
	Non-white	29.9	22.2	< 0.001	12.7	8.6	0.015
Married or living with a partner	Yes	25.4	18.8	< 0.001	8.2	5.2	0.073
	No	29.1	22.1	< 0.001	14.1	9.3	0.001
Per capita income	< 1 MW	24.5	20.1	0.013	12.1	7.9	0.031
	≥ 1 MW	29.0	20.8	< 0.001	11.8	7.7	0.003
Residence status	Urban	26.7	19.8	< 0.001	11.9	7.6	< 0.001
	Rural	33.1	25.0	< 0.001	11.6	9.5	0.521
Type of vehicle driven	Only car	24.3	17.6	< 0.001	10.0	7.4	0.071
	Motorcycle	29.5	23.1	< 0.001	14.8	8.4	< 0.001

^a^ p-value of the test of comparison of the prevalence in 2013 and 2019 (Pearson’s chi-squared test adjusted by the second-order Rao-Scott correction and converted into an F statistic).

Regarding residence status and the type of vehicle driven, for both men and women who lived in the urban area and were motorcycle drivers, the decrease was significant; for rural residents and car drivers, the decrease in prevalence was significant only for men. Regarding the type of vehicle driven, the decrease among motorcycle drivers was significant for both men and women. Among men who were car drivers, the decrease was significant (Table 3).

Table 4 shows the association between the habit of drinking and driving and the involvement of individuals in traffic accidents as car, van, or motorcycle drivers with bodily injuries in the last 12 months. ORs of involvement in traffic accidents were significantly higher among men and women who had the habit of drinking and driving. In 2013 and 2019, crude and adjusted ORs for men were close to 2. For women, in 2013, ORs were higher than 4 and in 2019, 2.7.

**Table 4:** Association between the habit of drinking and driving and the involvement of individuals in traffic accidents as car, van, or motorcycle drivers with bodily injuries in the last 12 months. PNS, Brazil, 2013 and 2019.

Drank and drove	2013										2019									
	
	n	%	95%CI		OR	95%CI		OR_adj_	95%CI		n	%	95%CI		OR	95%CI		OR_adj_	95%CI	
					
			LL	UL		LL	UL		LL	UL			LL	UL		LL	UL		LL	UL
**Men**	**499**	**5.7**	**4.9**	**6.7**	-	-	-	-	-	-	**777**	**4.4**	**3.9**	**5.0**	-	-	-	-	-	-
Yes	196	8.2	6.4	10.4	1.77ª	1.27	2.47	1.74ª	1.24	2.44	253	7.0	5.6	8.7	1.91ª	1.44	2.54	1.80ª	1.35	2.40
No	303	4.8	3.9	5.9	1.00	-	-	1.00	-	-	524	3.8	3.2	4.4	1.00	-	-	1.00	-	-
**Women**	**77**	**3.6**	**2.5**	**5.2**	-	-	-	-	-	-	**199**	**3.0**	**2.3**	**4.1**	-	-	-	-	-	-
Yes	28	11.0	6.0	19.5	4.62ª	2.27	9.40	4.99ª	2.26	11.00	35	6.9	3.4	13.7	2.76ª	1.20	6.38	2.73ª	1.21	6.19
No	49	2.6	1.7	4.1	1.00	-	-	1.00	-	-	164	2.7	1.9	3.8	1.00	-	-	1.00	-	-

OR: crude odds ratio; OR_adj_: odds ratio adjusted by the variables age group, married or living with a partner, and *per capita* income.

^a^ p ≤ 0.05.

Values for men and women are shown in bold.

## DISCUSSION

This study showed that, according to information from the PNS 2013 and 2019, the prevalence of drinking and driving decreased among the Brazilian population. In both editions of the PNS, the highest prevalence of drinking and driving was among men when compared with women. Individuals aged 30 to 39 years, who lived without a partner, in rural areas, and were motorcycle drivers had significantly higher estimates.

Men with higher *per capita* income presented higher prevalence of drinking and driving, corroborating the results of other studies^13,14^. The greater purchasing power to buy a motor vehicle and consume alcohol often is a possible explanatory hypothesis^2^. A Brazilian study with individuals who had left parties and nightclubs showed that the family income of those who drank and drove was higher than eight minimum wages^15^.

Our results showed higher prevalence of drinking and driving in rural areas. In Australia, a study also showed that this habit was more frequent among individuals living in rural areas and most drunk drivers were men. The authors state that the motivations for this practice in rural areas are different from those in urban areas, thus, more research and surveillance is needed^16^. A study in Montana, a rural area of the United States, showed that long and empty roads, few transportation alternatives, and lack of surveillance could explain the high prevalence of drinking and driving in these regions^17^. When comparing countries, however, considering the cultural and socioeconomic characteristics and the different contexts in which this behavior is more frequent is necessary^18^.

Regarding the type of vehicle driven, according to the two editions of the PNS, the prevalence of drinking and driving was significantly higher among motorcycle drivers. A study with patients with traumatic injuries treated in an emergency service in São Paulo showed that half of the injuries resulting from traffic accidents occurred among motorcycle drivers^19^. Moreover, a study with motorcycle drivers involved in accidents and hospitalized in the trauma section of a hospital in Recife, Pernambuco, showed that 32.9% of motorcycle drivers consumed alcohol before the accident and driving after drinking was associated with excessive speed, non-wearing of the helmet, and individuals not having a license to drive motorcycles^20^, showing the need for greater educational interventions aimed at this type of driver.

The findings of this study showed a significant decrease in the habit of drinking and driving from 2013 to 2019. A study in the United States also showed that the prevalence of this habit significantly decreased due to national efforts and the implementation of a program to prevent this behavior^21^.

As previously discussed^2^, in this study, we found evidence of the association between traffic accidents and the habit of drinking and driving for both men and women, showing the importance of recognizing that consuming alcohol before driving can cause accidents.

As a strategy to reduce traffic accidents, the United Nations (UN) established that the period from 2021 to 2030 would be the new decade of action for road safety, in order to reduce traffic deaths and injuries by 50%^22^. The prevention of traffic accidents due to alcohol consumption is also in the UN Agenda 2030, which aims to achieve a sustainable development related to health and well-being, with goals to reduce the harmful alcohol use and road deaths and injuries^23^.

In Brazil, during the last decade, the *Projeto Vida no Trânsito* (PVT – Life in Traffic Project), an initiative coordinated by the Ministry of Health in conjunction with the Pan American Health Organization (PAHO), aimed to intervene in two main factors: excessive speed and drinking and driving^24^. Admittedly, inspections by sobriety checkpoints are fundamental strategies to reduce traffic mortality associated with this habit^25^. Scientific evidence based on studies performed in several countries reinforces that legal measures regulating blood alcohol level are essential to effectively reduce damage caused by traffic accidents^26^.

We must highlight some aspects about the attitudes of drivers towards this harmful behavior. Data from research conducted on the Internet in Australia showed that individuals who had this habit were less likely to agree that drinking and driving led to a higher risk of traffic accidents and had the perception that they could get away with surveillance. On the other hand, drivers who did not have this habit believed that surveillance strategies were too lenient. According to this research, drivers who neglect surveillance laws are part of a vulnerable and worrying group regarding the problem of drinking and driving^27^.

In Brazil, the implementation of the *Lei Seca* (Dry Law) in 2008 and its updating in 2012 represented an important advance in regulatory issues related to the habit of drinking and driving. Individuals who break the law, driving under the influence of alcohol, besides being fined and having their vehicle withheld and driver’s license suspended, need to undergo a 30-hour refresher course with modules that include classes on defensive driving.

Besides awareness campaigns, strict and continuous law enforcement is essential as a determining factor to create models of safe behavior on roads. The implementation of intersectoral policies and effective operations are, in fact, the main elements for a real change in traffic behavior, resulting from a perception of vulnerability and understanding of the risk based on rules that regulate and control this type of social behavior^28,29^.

Medeiros^30^ also states that the responsibility to risk behaviors is transferred to individuals to the detriment of a collective logic of production and consumption based on the interests of economic agents who use the media as their means of dissemination for profit. According to this author^30^, this logic overvalues the freedom of choice of the consumer citizen and exempts the alcohol industry from its responsibility in the face of the costs and losses caused by traffic accidents.

This study had limitations. Despite the large sample in both editions of the PNS, the number of women who are drivers and reported to have drunk before driving is small, thus, the significance levels of the tests may have been non-significant due to the sample size. As this study was cross-sectional, temporality and causality may be compromised.

Moreover, in the analysis of the association between the habit of drinking and driving and the involvement of individuals in traffic accidents, we could not know if these individuals were under the influence of alcohol; therefore, our findings should be evaluated considering this limitation. The PNS considers only traffic accidents with bodily injuries, which may underestimate the occurrence of such an event.
