# Supplementary material for: The habit of drinking and driving in Brazil: National Survey of Health 2013 and 2019
Source: Rev Saude Publica. 2022 Nov 18;56:115. doi: 10.11606/s1518-8787.2022056004472 (PMC9749658; doi:10.11606/s1518-8787.2022056004472)
Supplement: Translation

# Translation: O hábito de beber e dirigir no Brasil: Pesquisa Nacional de Saúde, 2013 e 2019

**Authors:** Lucas Sisinno Ribeiro, Giseli Nogueira Damacena, Paulo Roberto Borges de Souza, Célia Landmann Szwarcwald

**Affiliations:** I Brasil Fundação Oswaldo Cruz. Instituto de Comunicação e Informação Científica e Tecnológica em Saúde. Programa de Pós-Graduação em Informação e Comunicação em Saúde. Rio de Janeiro, RJ, Brasil; II Brasil Fundação Oswaldo Cruz. Instituto de Comunicação e Informação Científica e Tecnológica em Saúde. Laboratório de Informações em Saúde. Rio de Janeiro, RJ, Brasil

**Keywords:** Acidentes de Trânsito, prevenção & controle, Dirigir sob a Influência, Consumo Excessivo de Bebidas Alcoólicas, Fatores de Risco, Inquéritos Epidemiológicos

## Abstract

**OBJETIVO:**

Investigar os fatores associados ao hábito de beber e dirigir, bem como estimar as variações nas prevalências desse comportamento entre os anos de 2013 e 2019, por meio das informações das duas edições da Pesquisa Nacional de Saúde (PNS).

**MÉTODOS:**

A PNS é um estudo transversal, de âmbito nacional e base domiciliar. Nos anos de 2013 e 2019, foram entrevistados, respectivamente, 60.202 e 85.854 indivíduos. Para investigar a associação entre o indicador “beber e dirigir” e as variáveis do estudo, as razões de chances (RC) brutas e ajustadas foram estimadas por meio de modelos de regressão logística. Para a comparação das prevalências entre os anos estudados, foi utilizado o teste Qui-Quadrado de Pearson ajustado pela correção de Rao-Scott (que leva em consideração o efeito do plano de amostragem) e convertido em uma estatística F, testada no nível de significância de 5%.

**RESULTADOS:**

A prevalência do hábito de beber e dirigir foi maior entre os homens no ano de 2013 (27,4%; IC95% 25,6–29,3%) e no ano de 2019 (20,5%; IC95% 19,4–21,7%) do que entre as mulheres (11,9%; IC95% 9,9–14,2% e 7,2%; IC95% 6,7–9,0%, respectivamente). Estimativas significativamente mais altas foram apresentadas por pessoas de 30 anos a 39 anos, que vivem sem companheiro(a), residentes em áreas rurais e condutores de motocicleta. Maiores prevalências de beber e dirigir foram encontradas entre homens que possuem maior rendimento. Entre os anos de 2013 e 2019, foi observado um decréscimo significativo no ato de beber e dirigir. Quanto ao envolvimento em acidentes de trânsito, as RC foram significativas (p < 0,01) nos anos estudados em ambos os sexos.

**DISCUSSÃO:**

Os resultados mostram a necessidade de dar continuidade às políticas de fiscalização de alcoolemia e educação no trânsito, com ações específicas dirigidas às áreas rurais e aos condutores de motocicletas.

## INTRODUÇÃO

O álcool é uma droga lícita amplamente difundida que vem sendo utilizada em diferentes culturas há muitos séculos, associado às festividades, cerimônias religiosas e celebrações^1^. Em função de suas características psicotrópicas, seu uso favorece a sociabilidade e a integração entre os indivíduos e é, atualmente, um hábito social comum e frequente. Entretanto, apesar do álcool ser uma droga lícita, determinados padrões de consumo podem gerar consequências prejudiciais para a saúde do indivíduo e para a sociedade^2^.

Entre os problemas associados ao uso abusivo de álcool, estão os distúrbios mentais e comportamentais, incluindo a dependência, doenças crônicas não transmissíveis, como a cirrose hepática, alguns tipos de câncer e doenças cardiovasculares, bem como lesões resultantes de violência e acidentes de trânsito^3^. Segundo a Organização Mundial da Saúde (OMS), o consumo nocivo de álcool está associado a mais de três milhões de mortes por ano no mundo^4^. No Brasil, dados do Global Burden of Diseases (GBD) mostram que o consumo de álcool ocupou a 5^ª^ posição, em 2017, entre os fatores de risco que mais contribuem para o número total de Anos de Vida Perdidos Ajustados por Incapacidade^5,6^.

Tendo em vista os efeitos dessa substância no organismo, dirigir após consumi-la é considerado uma das principais causas de envolvimento em acidentes de trânsito^5^. As modificações sensoriais provocadas pela intoxicação alcoólica comprometem as habilidades psicomotoras do indivíduo, provocando riscos ao condutor, aos passageiros e aos pedestres^1,7^.

Segundo dados do Observatório de Saúde Global da OMS, no ano de 2019, o Brasil possuía uma taxa estimada de mortalidade no trânsito de 16,0 (por 100.000 habitantes), bem superior à encontrada em países desenvolvidos, como a Austrália (4,9 por 100.000 habitantes) e o Canadá (5,3 por 100.000 habitantes), contudo próxima a de outros países emergentes, como a China (17,4 por 100.000 habitantes)^8^. Nesse contexto, o monitoramento da alcoolemia tem um papel fundamental no que diz respeito à prevenção dos acidentes de trânsito no país^5,2^.

Nos Estados Unidos, dados do Centro de Administração Nacional de Segurança no Trânsito Rodoviário mostraram que, em 2016, 10.497 pessoas morreram em acidentes de trânsito causados pelo álcool, representando 28% de todas as mortes relacionadas ao trânsito^9^. No Brasil, informações do Departamento Nacional de Infraestrutura de Transportes (DNIT) mostraram que a Polícia Rodoviária Federal, no ano de 2017, flagrou 19.083 motoristas que dirigiram após consumir bebida alcoólica e que, no mesmo ano, 6.450 mil (33,8%) acidentes nas rodovias federais foram causados por motoristas alcoolizados, gerando 13 mil vítimas e cerca de mil mortes^10^.

Em estudo baseado em dados da Pesquisa Nacional de Saúde - 2013, verificou-se que 24,4% dos brasileiros tinham o hábito de beber e dirigir, que esse comportamento estava associado ao maior risco de envolvimento em acidentes de trânsito e que os condutores do sexo masculino, da faixa de idade de 18 a 39 anos, tinham o maior risco de dirigir sob efeito do álcool^2^.

Dados do inquérito de Vigilância de Fatores de Risco e Proteção para Doenças Crônicas por Inquérito Telefônico (Vigitel) do mesmo ano mostraram que 29,3% dos homens e 16,5% das mulheres residentes em municípios de capitais tinham o hábito de beber e dirigir^1^. Estudo recente com dados do Vigitel evidenciou redução no indicador de beber e dirigir entre os anos de 2007 e 2018^5^.

Na segunda edição da Pesquisa Nacional de Saúde (PNS), realizada em 2019, as questões referentes ao hábito de beber e dirigir foram repetidas, possibilitando estimar a variação temporal desse comportamento na população brasileira.

O objetivo desse estudo é investigar os fatores associados ao beber e dirigir, além de estimar as variações nas prevalências desse comportamento entre os anos de 2013 e 2019, por meio de informações das duas edições da PNS.

## MÉTODOS

Trata-se de um estudo transversal que utilizou como fonte os dados das edições de 2013 e 2019 da PNS.

A PNS é um inquérito de âmbito nacional e base domiciliar realizado pelo Ministério da Saúde em parceria com o Instituto Brasileiro de Geografia e Estatística (IBGE), nos anos de 2013 e 2019. A PNS teve aprovação da Comissão Nacional de Ética em Pesquisa (Conep), em julho de 2013, sob o número n^º^ 328.159, para a edição de 2013, e em agosto de 2019, sob o número n^º^ 3.529.376, para a edição de 2019.

### Amostragem

A população pesquisada corresponde aos moradores de domicílios particulares permanentes no Brasil, exceto os localizados nos setores censitários especiais.

A amostra da PNS constitui uma subamostra da Amostra Mestra do Sistema Integrado de Pesquisas Domiciliares (SIPD) do IBGE. O plano amostral foi realizado por meio de conglomerados em três estágios de seleção (setores censitários ou composição de setores, domicílios, indivíduos) com estratificação das unidades primárias de amostragem (UPA). O detalhamento do processo amostral e do cálculo dos fatores de expansão encontra-se na publicação de Souza Jr^11^.

Na edição de 2013, foram selecionados 60.202 indivíduos de 18 anos ou mais para a entrevista individual; na edição de 2019, o número foi de 85.854 indivíduos.

### Variáveis do Estudo

Foram utilizadas as informações do questionário individual, respondido por um morador de 18 anos ou mais selecionado no domicílio.

Para a construção do indicador “beber e dirigir”, utilizou-se a seguinte pergunta nas duas edições da PNS: “Em algum destes dias em que consumiu bebida alcoólica, o(a) sr(a) dirigiu logo depois de beber?”. Com as seguintes opções de respostas: 1) Sim; 2) Não. Essa pergunta foi feita apenas aos indivíduos que informaram consumir bebida alcoólica e que dirigem automóvel ou motocicleta. Os condutores foram identificados na pesquisa a partir das seguintes perguntas: “Atualmente, o(a) Sr(a) dirige automóvel (inclusive táxi, aplicativos de transportes e similares)?” e “Atualmente, o(a) Sr(a) dirige motocicleta?” (Figura). O referido indicador foi analisado segundo características sociodemográficas: sexo (masculino; feminino); faixa etária (18–29 anos; 30–39 anos; 40 anos ou mais); cor da pele/raça (branca; não branca); casado(a) ou vive com companheiro(a) (sim; não), renda per capita (< 1 salário-mínimo (SM); ≥ 1 SM); situação urbano/rural do domicílio de residência; e tipo de veículo conduzido (apenas carro e motocicleta).

**Figura:** Fluxo de perguntas para captar os indivíduos que têm o hábito de beber e dirigir. PNS, Brasil, 2013 e 2019.

### Análise de Dados

Para cada uma das categorias das variáveis consideradas no estudo, estimou-se a prevalência do indicador “beber e dirigir” e seus respectivos intervalos de 95% de confiança em 2013 e em 2019. Para investigar a associação entre esse indicador e as variáveis de estudo, as razões de chances (RC) brutas e ajustadas por todas as demais variáveis foram estimadas por meio de modelos de regressão logística.

Para a comparação das prevalências entre os anos de 2013 e 2019, foi utilizado o teste qui-quadrado de Pearson ajustado pela correção de segunda ordem de Rao-Scott (que leva em consideração o efeito do plano de amostragem) e convertido em uma estatística F, testada no nível de significância de 5%.

Ademais, foi investigado, consoante o sexo, o envolvimento em acidente de trânsito como condutor(a) de carro, van ou motocicleta com lesões corporais nos últimos 12 meses a partir da pergunta: “Nos últimos 12 meses, o(a) sr(a) se envolveu em algum acidente de trânsito no qual tenha sofrido lesões corporais (ferimentos)?”. Para investigar a associação entre o indicador “beber e dirigir” e o envolvimento em acidente de trânsito nos últimos 12 meses, utilizou-se modelos de regressão logística por sexo. Foram estimadas as RC brutas e ajustadas por faixa de idade, casado(a) ou vive com companheiro(a) e renda per capita, sendo testadas no nível de significância de 5%.

A análise estatística levou em consideração o desenho amostral das duas pesquisas, incluindo os fatores de expansão e os efeitos de conglomeração. Os dados foram analisados utilizando o *Software for Statistics and Data Science*^12^, versão 14.0, módulo “*survey*”.

## RESULTADOS

Foram analisados 2.651 indivíduos em 2013 e 4.114 em 2019, os quais dirigiram depois de beber. A prevalência do hábito de beber e dirigir foi maior entre os homens nos dois anos pesquisados (27,4%, IC95% 25,6–29,3%, em 2013; 20,5%, IC95% 19,4–21,7%, em 2019) do que entre as mulheres (11,9%, IC95% 9,9–14,2%, em 2013; 7,2%, IC95% 6,7–9,0%, em 2019) (Tabelas 1 e 2). Esse comportamento foi mais prevalente na faixa de 30 a 39 anos tanto para o sexo masculino quanto para o feminino, nas duas edições da PNS.

**Tabela 1:** Prevalências do hábito de beber e dirigir e razões de chances (brutas e ajustadas) segundo variáveis sociodemográficas, situação de residência e tipo de veículo conduzido entre indivíduos do sexo masculino com 18 anos ou mais de idade. PNS, Brasil, 2013 e 2019.

Variáveis		2013										2019									
	
		n	%	IC95%		RC	IC95%		RC_aj_	IC95%		n	%	IC95%		RC	IC95%		RC_aj_	IC95%	
					
				LI	LS		LI	LS		LI	LS			LI	LS		LI	LS		LI	LS
Total		2.397	27,4	25,6	29,3	-	-	-	-	-	-	3.539	20,5	19,4	21,7	-	-	-	-	-	-
Faixa de idade	18–29	779	29,7	26,3	33,2	1,39ª	1,13	1,70	1,29^a^	1,02	1,63	966	23,5	21,2	26,0	1,51ª	1,28	1,80	1,44^a^	1,20	1,73
	30–39	740	31,4	28,0	35,1	1,51ª	1,22	1,86	1,45^a^	1,17	1,80	1.123	24,7	22,4	27,2	1,62ª	1,35	1,93	1,56^a^	1,30	1,87
	≥ 40	878	23,3	20,9	25,9	1,00	-	-	1,00	-	-	1.449	16,9	15,3	18,6	1,00	-	-	1,00	-	-
Raça/cor da pele	Branca	1.191	25,5	23,2	28,1	1,00	-	-	1,00	-	-	1.574	18,9	17,4	20,5	1,00	-	-	1,00	-	-
	Não branca	1.188	29,9	27,2	32,6	1,24^a^	1,05	1,47	1,24^a^	1,04	1,49	1.965	22,2	20,4	24,0	1,22^a^	1,06	1,42	1,16	0,99	1,37
Casado(a) ou vive com companheiro(a)	Sim	1.005	25,4	23,0	28,0	1,00	-	-	1,00	-	-	1.554	18,8	17,1	20,7	1,00	-	-	1,00	-	-
	Não	1392	29,1	26,6	31,7	1,21ª	1,02	1,42	1,07	0,88	1,28	1.985	22,1	20,7	23,6	1,22ª	1,06	1,41	1,03	0,88	1,21
Renda per capita	< 1 SM	764	24,5	21,7	27,5	1,00	-	-	1,00	-	-	1.360	20,1	18,5	21,8	1,00	-	-	1,00	-	-
	≥ 1 SM	1.631	29,0	26,8	31,4	1,26ª	1,05	1,51	1,57^a^	1,28	1,92	2.179	20,8	19,3	22,4	1,05	0,92	1,19	1,35^a^	1,15	1,57
Situação de residência	Urbana	2.063	26,7	24,7	28,8	1,00	-	-	1,00	-	-	2901	19,8	18,5	21,1	1,00	-	-	1,00	-	-
	Rural	333	33,1	28,9	37,6	1,36ª	1,09	1,69	1,47^a^	1,16	1,86	638	25,0	22,9	27,3	1,36ª	1,18	1,56	1,34^a^	1,15	1,57
Tipo de veículo conduzido	Apenas carro	860	24,3	21,6	27,1	1,00	-	-	1,00	-	-	1.421	17,6	16,2	19,1	1,00	-	-	1,00	-	-
	Motocicleta	1.537	29,5	27,2	32,1	1,31^a^	1,09	1,57	1,20	0,98	1,48	2.118	23,1	21,5	24,8	1,40^a^	123	1,60	1,28^a^	1,09	1,51

IC95%: intervalo de confiança de 95%; LI: limite inferior do intervalo de confiança; LS: limite superior do intervalo de confiança; SM: salário-mínimo; RC: razão de chances bruta; RC_aj_: razão de chances ajustada pelas demais variáveis.

^a^ valor de p ≤ 0,05.

**Tabela 2:** Prevalências do hábito de beber e dirigir e razões de chances (brutas e ajustadas) segundo variáveis sociodemográficas, situação de residência e tipo de veículo conduzido entre indivíduos do sexo feminino com 18 anos ou mais de idade. PNS, Brasil, 2013 e 2019.

Variáveis		2013										2019									
	
		n	%	IC95%		RC	IC95%		RC_aj_	IC95%		n	%	IC95%		RC	IC95%		RC_aj_	IC95%	
								
				LI	LS		LI	LS		LI	LS			LI	LS		LI	LS		LI	LS
Total		255	11,9	9,9	14,2	-	-	-	-	-	-	506	7,8	6,7	9,0	-	-	-	-	-	-
Faixa de idade	18–29	86	10,7	7,8	14,6	1,19	0,76	1,85	0,82	0,52	1,29	126	7,2	5,1	9,9	1,20	0,78	1,84	1,03	0,65	1,63
	30–39	103	16,3	12,3	21,4	1,92^a^	1,28	2,89	1,78^a^	1,15	2,77	212	10,8	8,6	13,4	1,89^a^	1,34	2,63	1,88^a^	1,32	2,69
	≤ 40	66	9,2	6,6	12,6	1,00	-	-	1,00	-	-	168	6,0	4,9	7,4	1,00	-	-	1,00	-	-
Raça/cor da pele	Branca	153	11,4	9,0	14,3	1,00	-	-	1,00	-	-	284	7,1	5,8	8,8	1,00	-	-	1,00	-	-
	Não branca	99	12,7	9,8	16,5	1,14	0,80	1,61	0,90	0,62	1,29	214	8,6	7,0	10,4	1,22	0,89	1,66	1,13	0,79	1,62
Casado(a) ou vive com companheiro(a)	Sim	66	8,2	5,5	11,9	1,00	-	-	1,00	-	-	129	5,2	3,9	7,1	1,00	-	-	1,00	-	-
	Não	188	14,1	11,6	17,1	1,85^a^	1,20	2,84	2,09^a^	1,38	3,17	377	9,3	7,9	11,0	1,86^a^	1,28	2,70	1,94^a^	1,32	2,84
Renda per capita	< 1 SM	65	12,1	8,8	16,5	1,00	-	-	1,00	-	-	142	7,9	6,1	10,1	1,00	-	-	1,00	-	-
	≥ 1 SM	189	11,8	9,5	14,5	0,97	0,67	1,40	1,28	0,84	1,95	364	7,7	6,5	9,2	0,98	0,70	1,37	1,24	0,87	1,78
Situação de residência	Urbana	239	11,9	9,8	14,3	1,00	-	-	1,00	-	-	458	7,6	6,5	8,9	1,00	-	-	1,00	-	-
	Rural	15	11,6	6,4	20,2	0,98	0,66	1,43	0,92	0,59	1,44	48	9,5	6,4	13,9	1,28	0,80	2,04	1,25	0,77	2,05
Tipo de veículo conduzido	Apenas carro	132	10,0	7,7	12,9	1,00	-	-	1,00	-	-	318	7,4	6,2	9,0	1,00	-	-	1,00	-	-
	Motocicleta	122	14,8	11,6	18,8	1,57^a^	1,08	2,27	1,68^a^	1,10	2,56	188	8,4	6,7	10,5	1,14	0,83	1,56	1,04	0,73	1,47

IC95%: intervalo de confiança de 95%; LI: limite inferior do intervalo de confiança; LS: limite superior do intervalo de confiança; SM: salário-mínimo; RC: razão de chances bruta; RC_aj_: razão de chances ajustada pelas demais variáveis.

^a^ valor de p ≤ 0,05.

Em 2013, homens entre 18 e 29 anos e entre 30 e 39 anos apresentaram razões de chance (RC) de 1,3 e 1,5 quando comparados aos de 40 anos ou mais. Em 2019, as RC foram, respectivamente, de 1,5 e 1,6, sendo significativas mesmo após os ajustes pelas demais variáveis (Tabela 1).

O hábito de beber e dirigir apresentou maiores prevalências entre os homens de raça/cor de pele não branca. Tanto em 2013 como em 2019, homens não brancos possuíam chance significativamente maior de beber e dirigir em comparação com os brancos. Entretanto, após o controle das demais variáveis, a RC foi significativa apenas para o ano de 2013 (Tabela 1).

Nos dois anos pesquisados, o beber e dirigir foi mais frequente entre os que não são casados ou que não vivem com companheira(o). A chance disso acontecer foi 20% maior entre os homens que vivem sem companheiro(a), porém as RC não foram significativas, após os ajustes pelas demais variáveis (Tabela 1).

No que diz respeito à renda per capita, o hábito aqui analisado foi maior entre os homens cujo rendimento é ≥ 1 SM. Comparados aos que têm renda < 1 SM, em 2013, as RC bruta e ajustada foram estatisticamente significativas, de 1,4 e 1,5, respectivamente. Em 2019, apenas a RC ajustada foi significativa, indicando que, após o controle por faixa de idade, esse comportamento foi mais prevalente entre os de maior renda per capita (Tabela 1).

Quanto à situação do domicílio (urbano/rural), os homens que viviam na área rural tiveram maiores prevalências do hábito de beber e dirigir. Comparando com os residentes em área urbana, tanto as RC brutas como as ajustadas foram significativas nos dois anos pesquisados (Tabela 1).

Em relação ao tipo de veículo conduzido, foi possível observar que os homens que dirigiam motocicleta tiveram RC brutas significativamente > 1 em comparação aos que dirigiam apenas carro nos dois anos considerados no estudo. Após o controle das demais variáveis, a RC foi significativa apenas em 2019 (Tabela 1).

Na Tabela 2 são apresentados os resultados para as mulheres. O hábito de beber e dirigir foi mais prevalente entre aquelas que tinham entre 30 a 39 anos, em 2013 e 2019. Em comparação às mulheres com 40 anos ou mais, as RC brutas e ajustadas foram significativas, variando de 1,7 a 1,9. Comparadas às que têm de 30 a 39 anos, os resultados relativos às mulheres de 18 a 29 anos não foram estatisticamente significativos.

De forma semelhante aos homens, mulheres que não eram casadas ou viviam sem companheiro(a) tiveram as maiores prevalências em relação ao ato de beber e dirigir. Nos anos de 2013 e 2019, quando comparadas às que viviam com companheiro(a), as RC brutas foram 1,8, e as ajustadas, 2,2 e 1,9, respectivamente, significativas no nível de 5% (Tabela 2).

Em relação ao tipo de veículo conduzido, mulheres que dirigiam motocicleta apresentaram maiores prevalências do comportamento analisado apenas em 2013. Nesse ano, comparadas às mulheres que dirigiam apenas carro, as RC bruta e ajustada foram significativas, de 1,6 e 1,7 (Tabela 2). Não foram encontradas diferenças evidentes no hábito de beber e dirigir segundo a cor da pele nem segundo rendimento per capita (Tabela 2).

Na tabela 3, são comparadas as prevalências desse hábito entre 2013 e 2019. Houve uma diminuição significativa da prevalência de 27,4% para 20,5% entre os homens e de 11,9% para 7,8% entre as mulheres. Quanto às variações por grupo de idade, entre os homens, reduções significativas ocorreram em todas as faixas; entre as mulheres, nas faixas de 30 a 39 anos e de 40 anos ou mais (Tabela 3). No que diz respeito à cor da pele, foi possível verificar decréscimos significativos tanto entre os brancos como entre os não brancos para ambos os sexos. Diminuições significativas no beber e dirigir foram encontradas também entre aqueles que não são casados(as) ou não vivem com companheiros(as). Entre os indivíduos casados ou que vivem com companheiros(as), essa redução foi significativa somente entre os homens (Tabela 3). A análise por renda per capita mostrou diminuições significativas do beber e dirigir para as duas categorias entre ambos os sexos.

**Tabela 3:** Comparação das prevalências do hábito de beber e dirigir entre 2013 e 2019 segundo as características sociodemográficas e tipo de veículo conduzido. PNS, Brasil, 2013 e 2019.

Variáveis		Masculino			Feminino		
	
		% 2013	% 2019	p^a^	% 2013	% 2019	p^a^
Total		27,4	20,5	< 0,001	11,9	7,8	< 0,001
Faixa de idade (anos)	18–29	29,7	23,5	0,003	10,7	7,2	0,080
	30–39	31,4	24,7	0,002	16,3	10,8	0,023
	≥ 40	23,3	16,9	< 0,001	9,2	6,0	0,031
Raça/cor da pele	Branca	25,5	18,9	< 0,001	11,4	7,1	0,003
	Não branca	29,9	22,2	< 0,001	12,7	8,6	0,015
Casado(a) ou vive com companheiro(a)	Sim	25,4	18,8	< 0,001	8,2	5,2	0,073
	Não	29,1	22,1	< 0,001	14,1	9,3	0,001
Renda per capita	< 1 SM	24,5	20,1	0,013	12,1	7,9	0,031
	≥ 1 SM	29,0	20,8	< 0,001	11,8	7,7	0,003
Situação de residência	Urbana	26,7	19,8	< 0,001	11,9	7,6	< 0,001
	Rural	33,1	25,0	< 0,001	11,6	9,5	0,521
Tipo de veículo conduzido	Apenas carro	24,3	17,6	< 0,001	10,0	7,4	0,071
	Motocicleta	29,5	23,1	< 0,001	14,8	8,4	< 0,001

^a^ Valor de p do teste de comparação das prevalências entre 2013 e 2019 (qui-quadrado de Pearson ajustado pela correção de segunda ordem de Rao-Scott e convertido em uma estatística F).

Quanto à situação urbano/rural e ao tipo de veículo conduzido, houve redução significativa para aqueles que residiam na área urbana e que eram condutores de motocicleta para os dois sexos; para os residentes da área rural e condutores somente de carro, a diminuição nas prevalências foi significativa apenas para os homens. No que se refere ao tipo de veículo conduzido, o decréscimo entre aqueles que eram condutores de motocicleta foi significativo, para ambos os sexos. Para os que eram condutores somente de carro, houve redução significativa entre os homens (Tabela 3).

Na Tabela 4, apresenta-se a análise da associação entre o hábito de beber e dirigir e o envolvimento em acidente de trânsito como condutor(a) de carro, van ou motocicleta com lesões corporais nos últimos 12 meses. Foi possível observar que as RC de envolvimento em acidentes de trânsito foram significativamente maiores entre homens e mulheres que tinham o hábito de beber e dirigir. Em 2013 e 2019, as RC brutas e ajustadas para o sexo masculino se aproximaram de 2. Para o sexo feminino, em 2013, as RC foram superiores a 4; em 2019, 2,7.

**Tabela 4:** Associação entre o hábito de beber e dirigir e o envolvimento em acidente de trânsito como condutor(a) de carro, van ou motocicleta com lesões corporais nos últimos 12 meses e o hábito de beber e dirigir. PNS, Brasil, 2013 e 2019.

Bebeu e dirigiu	2013										2019									
	
	n	%	IC95%		RC	IC95%		RC_aj_	IC95%		n	%	IC95%		RC	IC95%		RC_aj_	IC95%	
					
			LI	LS		LI	LS		LI	LS			LI	LS		LI	LS		LI	LS
Masculino	**499**	**5,7**	**4,9**	**6,7**	-	-	-	-	-	-	**777**	**4,4**	**3,9**	**5,0**	-	-	-	-	-	-
Sim	196	8,2	6,4	10,4	1,77ª	1,27	2,47	1,74ª	1,24	2,44	253	7,0	5,6	8,7	1,91ª	1,44	2,54	1,80ª	1,35	2,40
Não	303	4,8	3,9	5,9	1,00	-	-	1,00	-	-	524	3,8	3,2	4,4	1,00	-	-	1,00	-	-
Feminino	**77**	**3,6**	**2,5**	**5,2**	-	-	-	-	-	-	**199**	**3,0**	**2,3**	**4,1**	-	-	-	-	-	-
Sim	28	11,0	6,0	19,5	4,62ª	2,27	9,40	4,99ª	2,26	11,00	35	6,9	3,4	13,7	2,76ª	1,20	6,38	2,73ª	1,21	6,19
Não	49	2,6	1,7	4,1	1,00	-	-	1,00	-	-	164	2,7	1,9	3,8	1,00	-	-	1,00	-	-

RC: razão de chances bruta; RC_aj_: razão de chances ajustada por faixa de idade, casado(a) ou vive com companheiro(a) e renda per capita.

^a^ valor de p < 0,05.

Valores para o sexo masculino e feminino estão mostrados em negrito.

## DISCUSSÃO

Nesse artigo, por meio de informações coletadas na PNS-2013 e na PNS-2019, foi evidenciada uma redução da prevalência do beber e dirigir na população brasileira. Nas duas edições da PNS, as maiores prevalências do hábito de beber e dirigir foram observadas entre os homens, quando comparados às mulheres. Estimativas significativamente mais altas foram apresentadas por pessoas da faixa de idade de 30 a 39 anos, indivíduos que vivem sem companheiro(a) e condutores de motocicleta.

Maiores prevalências de beber e dirigir foram encontradas entre indivíduos do sexo masculino que possuem maior renda per capita, corroborando resultados de outros estudos^13,14^. O maior poder aquisitivo para adquirir um veículo automotor e consumir, habitualmente, bebidas alcoólicas é uma possível hipótese explicativa^2^. De fato, estudo brasileiro com pessoas que tinham saído de festas e casas noturnas mostrou que a renda familiar entre aqueles que beberam e dirigiram era superior a oito salários-mínimos^15^.

Os resultados deste artigo revelaram maiores prevalências do beber e dirigir nas áreas rurais. Na Austrália, um estudo mostrou, igualmente, que esse hábito era mais frequente entre aqueles que moravam em áreas rurais, e que a maior parte dos motoristas que dirigiam embriagados nesses locais eram do sexo masculino. Os autores do estudo ressaltam que as motivações para essa prática em áreas rurais são diferentes das de áreas urbanas, por isso são necessárias mais pesquisas, intervenções e fiscalizações^16^. Outro estudo, realizado em Montana, área rural dos Estados Unidos, mostrou que estradas longas e vazias, poucas alternativas de transporte e a falta de fiscalização poderiam explicar as altas prevalências do beber e dirigir nessas regiões^17^. Na comparação entre os países, entretanto, é preciso considerar as características culturais e socioeconômicas e os distintos contextos em que esse comportamento é mais frequente^18^.

No que se refere ao tipo de veículo conduzido, segundo as informações das duas edições da PNS, as prevalências do hábito de beber e dirigir foram significativamente maiores entre os condutores de motocicleta. Trabalho realizado com pacientes atendidos em um serviço de emergência com lesões traumáticas em São Paulo mostrou que metade das lesões resultantes de acidentes de trânsito ocorreram entre motociclistas^19^. Já estudo realizado com motociclistas envolvidos em acidentes e internados no setor de traumatologia de um hospital de Recife, Pernambuco, revelou que o consumo de álcool antes do acidente foi relatado por 32,9% dos motociclistas e que o comportamento de dirigir após beber foi associado com o excesso de velocidade, o não uso do capacete e o fato de não possuir habilitação para motocicleta^20^, mostrando a necessidade de maiores intervenções educativas voltadas para esse tipo de condutor.

Os achados desse estudo mostraram redução significativa do hábito de beber e dirigir entre os anos de 2013 e 2019. Pesquisa realizada nos Estados Unidos revelou, igualmente, que a prevalência desse hábito diminuiu significativamente em função de esforços nacionais e da implementação de programa de prevenção a esse comportamento^21^.

Conforme já discutido anteriormente^2^, evidências referentes à associação entre o envolvimento de acidente de trânsito e o hábito de beber e dirigir foram encontradas nesse trabalho, entre homens e mulheres, apontando para a importância de reconhecer o uso de álcool antes de dirigir como um fator acidentogênico.

Como estratégia para a diminuição dos acidentes de trânsito, a Organização das Nações Unidas (ONU) determinou que os anos entre 2021 e 2030 formariam a nova década de ação pela segurança no trânsito, a fim de diminuir em 50% as mortes e lesões no trânsito nesse período^22^. A prevenção de acidentes de trânsito causados em função do consumo de bebidas alcóolicas também está presente na Agenda 2030 da ONU, com o objetivo de realizar um desenvolvimento sustentável relacionado à saúde e ao bem-estar, com metas para a redução do uso nocivo de álcool e das mortes e ferimentos em estradas^23^.

No Brasil, na última década, o projeto “Vida no Trânsito”, uma iniciativa coordenada pelo Ministério da Saúde em articulação com a Organização Pan Americana da Saúde (Opas), teve o objetivo de intervir em dois principais fatores: a velocidade excessiva e o beber e dirigir^24^. Reconhecidamente, as fiscalizações por *checkpoints* de sobriedade são estratégias fundamentais para reduzir a mortalidade no trânsito associada a esse hábito^25^. Evidências científicas baseadas em estudos realizados em diversos países reforçam que medidas legais que regulamentam a alcoolemia são essenciais para uma redução efetiva dos danos causados pelos acidentes de trânsito^26^.

É preciso ressaltar alguns aspectos sobre as atitudes dos motoristas perante esse comportamento nocivo. Dados de uma pesquisa realizada pela internet, na Austrália, mostrou que indivíduos que tinham esse comportamento eram menos propensos a concordar que o hábito de beber e dirigir levava a um maior risco de acidentes de trânsito, além da percepção de que poderiam escapar impunemente pela fiscalização. Em contraposição, motoristas que não tinham esse costume acreditavam que as estratégias de fiscalização eram muito brandas. A partir dessa pesquisa, pode-se inferir que os condutores que mostram descaso pelas leis de fiscalização se configuram como um grupo vulnerável e preocupante em relação ao problema do beber e dirigir^27^.

No Brasil, a implantação da “Lei Seca”, no ano de 2008, e sua atualização, em 2012, representaram um importante avanço nas questões regulatórias relacionadas ao hábito de beber e dirigir. Pessoas que infringem a lei dirigindo sob efeito do álcool, além de serem multadas, terem o veículo retido e a Carteira Nacional de Habilitação suspensa, precisam passar por um curso de reciclagem de 30 horas com módulos que incluem aulas sobre direção defensiva.

Além das campanhas de conscientização, é fundamental a aplicação rigorosa e contínua da lei como um fator determinante para criar modelos de comportamento seguro nas vias. A implementação de políticas intersetoriais e de operações efetivas são, de fato, os principais elementos para uma mudança real nos comportamentos no trânsito a partir de uma percepção de vulnerabilidade e conhecimento do risco baseada nas normas que regulam e controlam esse tipo de comportamento social^28,29^.

Medeiros^30^ ressalta ainda que a responsabilidade com os comportamentos de riscos é transferida para os indivíduos, em detrimento de uma lógica coletiva de produção e consumo baseada em interesses de agentes econômicos que utilizam a mídia como seu meio de difusão visando ao lucro. Segundo este autor^30^, essa lógica hipervaloriza a liberdade de escolha do cidadão-consumidor e isenta a indústria do álcool de sua responsabilidade frente aos custos e prejuízos gerados com os acidentes de trânsito.

Entre as limitações desse estudo, é importante ressaltar que, apesar da amostra grande em ambas as edições da PNS, o número de mulheres que são motoristas e responderam ter bebido antes de dirigir é pequeno, então níveis de significância dos testes podem ter sido não significativos devido ao tamanho da amostra. Além disso, devido à natureza transversal desse estudo, deve-se considerar que a temporalidade e a causalidade podem ficar comprometidas.

Ademais, na análise da associação entre o hábito de beber e dirigir e o envolvimento em acidentes de trânsito, não foi possível saber se o indivíduo que se envolveu em acidente de trânsito estava em estado de alcoolemia, portanto esses achados devem ser examinados à luz desta limitação. A PNS apenas considera acidentes de trânsito com lesões corporais, o que pode estar subestimando a ocorrência de tal evento.
